# Turning the Page to Year 2016

**DOI:** 10.5772/62174

**Published:** 2015-01-01

**Authors:** Markus Dettenhofer, Winston Patrick Kuo

**Affiliations:** 1 CEITEC Executive Director, Brno, Czech Republic; 2 Predicine Holdings Ltd, Hayward, CA, USA

## Abstract

As we conclude another year (2015), Volume 2 completed, we are pleased with the number of quality published manuscripts. We are also excited to announce Nanobiomedicine has been indexed in DOAJ (Directory of Open Access Journals) (https://doaj.org/toc/1849-5435)! This was in part attributed with the help of our Special Editor, Dr. Barbara Smith, who spearheaded manuscripts highlighting innovative results that impacted the global health spectrum implementing new methods for disease diagnosis, including technological and product development for enhanced point-of-care and personalized health care. Dr. Smith undertook this endeavor as she transitioned from a post-doc position (from George Whitesides' lab at Harvard University) to a faculty position at Arizona State, getting acclimated and setting up her laboratory. We want to thank Dr. Smith for her time and commitment to our journal. It's worth noting, we had a high number of submissions throughout the year, however, the expectations of the manuscripts not published fell short due to our review process, indicating the emphasis of publishing high quality manuscripts. We thank all the reviewers for their time and feedback.

## Nanobiomedicine in Numbers

By the end of its 2^nd^ volume, the journal's articles have been viewed more than 40,000 times and downloaded more than 6,000 times in total. The top 3 countries by number of submissions were USA, Czech Republic and Spain, while the most frequent readers were from India, Austria and USA. Based on the ratio between the number of views and downloads, the most popular research paper published in the journal for the year 2015 is the following:

ELISA-like Analysis of Cisplatinated DNA Using Magnetic Separation- by Kristyna Smerkova, Marcela Vlcnovska, Simona Dostalova, Vedran Milosavljevic, Pavel Kopel, Tomas Vaculovic, Sona Krizkova, Marketa Vaculovicova, Vojtech Adam and Rene Kizek.

This original research article was viewed more than 800 times in the two months since being published online.

On the other hand, the most downloaded paper of 2015 is another original research article titled:

Influence of Growth Parameters on the Formation of Hydroxyapatite (HAp) Nanostructures and Their Cell Viability Structures- by Murugesan Manoj, Ramesh Subbiah, Devanesan Mangalaraj, Nagamony Ponpandian, Chinnuswamy Viswanathan and Kwideok Park.

## The journal looks forward to 2016

The journal looks forward to 2016 with new initiatives, by increasing our efforts to reach out to academia and industry to submit their manuscripts around nanobiomedicine including, but not limited to the following areas; bioengineering, nano-technological applications in diagnostics, therapeutic application, vaccine, drug discovery/delivery and regenerative medicine. Also, given President Obama's call for $215 million in 2016 to support his Precision Medicine Initiative, this will enable a new era of medicine in which researchers, providers and patients will work together to develop individualized care. Precision medicine is an emerging approach for disease treatment and prevention, which exams each individual based on their variability in genes, environment, and lifestyle. $130 million of the allocated budget will be allocated to NIH to build a national, large-scale research participant group, called a cohort, and $70 million will be allocated to the National Cancer Institute to lead efforts in cancer genomics.

There are many similar precision medicine initiatives globally and given the field of nanobiomedicine, there are many opportunities in the area diagnostics, therapeutics and monitoring the response to therapy. So as a result of these ongoing initiatives, we will seek to partner with conference organizers not only in technical and application, but to the precision medicine topics, to play a role as their media partner, to publish abstracts and selected papers presented at their conferences.

Lastly, we have been actively engaged with the BioPharma Research Council (a partnership established last year) to co-host webinars related to the journal on a variety of topics and allow the presenter(s) to convert their presentation into a manuscript as part of a BioPharma Research Council/ Nanobiomedicine webinar series. One initiative in the process of being scheduled for April 2016 is a virtual conference around the theme of Point of Care Diagnostics. A committee with members from industry, including Thomas White, retired Chief Scientific Officer from Celera and, Joe Almeida from Siemens Healthcare have outlined a framework of relevant topics (lab-on-a-chip for sample preparation, detection systems including instruments and reagents, therapeutic applications to implementation (validation) and adoption) to be discussed during the one day (from 5–8 speakers) virtual conference.

## Volume 3 open for submissions on an ongoing basis

NBM accepts original and review articles as well as editorials, perspectives, short research reports, protocols and methods, notes to the editor, letters to the editor and meeting dispatch reports. The NBM manuscript processing and peer-review is entirely online. The Editorial Manager facilitates the manuscript processing time, reducing costs and is a better experience for our authors and reviewers. We have received numerous positive comments on this evolving electronic system, and we are indebted to InTech for managing this program for us. For more information, please visit our new manuscript submission system at http://www.intechopen.com/journals/show/nanobiomedicine/submit-your-paper. In addition, we are enforcing to expedite the acceptance to publishing time for our authors and will request informal opinions from our Editorial Board for borderline cases.

We would like to mention, InTech Open Access Publisher has decided that NBM will not apply any article processing charges for authors whose research papers have been accepted for publication in Volume 3/2016. Finally, we would like to thank InTech for supporting the initiatives of this journal and acknowledge Petra Nenadic and her team for her continuous and endless support, passion and dedication for making this possible. In addition, we would like to thank our highly renowned and respected Associate Editors and Editorial Board members from all over the world, whose expertise range from nano-fluidic systems to nano-scale drug delivery to nano-imaging that are bridge and a catalyst in their respective fields of nanotechnology and biomedical research.

